# Dose enhancement effects of gold nanoparticles specifically targeting RNA in breast cancer cells

**DOI:** 10.1371/journal.pone.0190183

**Published:** 2018-01-18

**Authors:** Georg Hildenbrand, Philipp Metzler, Götz Pilarczyk, Vladimir Bobu, Wilhelm Kriz, Hiltraud Hosser, Jens Fleckenstein, Matthias Krufczik, Felix Bestvater, Frederik Wenz, Michael Hausmann

**Affiliations:** 1 Kirchhoff-Institute for Physics, Faculty of Physics and Astronomy, Heidelberg University, Heidelberg, Germany; 2 Department of Radiation Oncology, Universitätsmedizin Mannheim, Medical Faculty Mannheim at Heidelberg University, Mannheim, Germany; 3 Department of Neuroanatomy, Center for Biomedicine and Medical Technology Mannheim (CBTM), Medical Faculty Mannheim at Heidelberg University, Mannheim, Germany; 4 Light Microscopy Faculty, German Cancer Research Centre (DKFZ), Heidelberg, Germany; Universidade Nova de Lisboa, PORTUGAL

## Abstract

Localization microscopy has shown to be capable of systematic investigations on the arrangement and counting of cellular uptake of gold nanoparticles (GNP) with nanometer resolution. In this article, we show that the application of specially modified RNA targeting gold nanoparticles (“SmartFlares”) can result in ring like shaped GNP arrangements around the cell nucleus. Transmission electron microscopy revealed GNP accumulation in vicinity to the intracellular membrane structures including them of the endoplasmatic reticulum. A quantification of the radio therapeutic dose enhancement as a proof of principle was conducted with γH2AX foci analysis: The application of both—SmartFlares and unmodified GNPs—lead to a significant dose enhancement with a factor of up to 1.2 times the dose deposition compared to non-treated breast cancer cells. This enhancement effect was even more pronounced for SmartFlares. Furthermore, it was shown that a magnetic field of 1 Tesla simultaneously applied during irradiation has no detectable influence on neither the structure nor the dose enhancement dealt by gold nanoparticles.

## Background

Recent years have seen a dramatic increase in interest regarding the use of gold nanoparticles (GNPs) as radiation sensitizers for radiotherapy [1]. This interest was initially driven by their high absorption of ionizing radiation and the resulting ability to increase the dose deposition within target volumes, even at low concentrations [2–4] leading to a significant RBE increase [3]. Since the field of radiotherapy is still struggling to spare normal tissue while enhancing the dose deposition in malignant cells [5], especially the directed transport of damage sources into cancer cells is becoming more and more the focus of attention. Since the absorption of a photoelectron by particles with high atomic number Z can lead to the release of several Auger electrons [1, 6–8], the position as well as the distribution of those particles is a crucial parameter when it comes to increasing the therapeutic window. High energetic photoelectrons are long-ranged (several cm in water), and therefore do not allow a precise and well calibrated dose deposition within a tumor without harming healthy tissue substantially. Auger electrons, on the other hand, have lower energy and therefore a shorter range (μm), thus leading to an energy deposition in proximity to their source [9–11].

The particle size plays a key role for a successful GNP deposition within cancer cells. Particles larger than 300 nm are potentially eliminated by macrophages, while GNPs smaller than 100 nm in diameter can enter the tumor tissue [12]. The optimum size of GNPs for cellular uptake and retention was found to be 50 nm [13]. GNPs smaller than 30 nm are leaving the cell again by passive diffusion [3, 14, 15]. However, the cellular uptake is only one side of the coin. Due to the necessary trade-off between the efficient enhancement of radiotherapy for small particles and an optimum cellular uptake the overall optimum particle size may be found at smaller diameters than 30 nm. Moser et al. [15] could show that 10 nm particles can be a good compromise between efficient enhancement of radio sensitivity for small particles such as used in Hainfeld’s studies [16–19] and an optimum cellular uptake for particles of larger diameters.

Hainfeld stated a significant increase in one-year survival (20% for x-rays alone versus 50–86% for x-rays with unmodified GNPs) of mice with tumors treated with 250 kV x-ray energy [16]. Other examples making use of passive targeting GNPs [17–19] showed dose enhancement factors of 1.17–1.66 [20–22], depending on the x-ray energy applied. This raises the important question, whether the use of specially modified GNPs that are aiming for specific parts of a cell would lead to a different GNP aggregation and distribution. The directed GNP deposition could have two enormous advantages over a non-targeting GNP application: Firstly, the GNP location and distribution might have a significant effect on the therapeutic gap. For example, a proximity of the nanogold cores to the cell nucleus might lead to a dose enhancement due to a higher number of double strand breaks (DBS) dealt by the low ranged Auger emitters. Secondly, the uptake could be discriminated between cancer cells and normal tissue by using targets that are predominantly existent in cancer cells but not in normal tissue possibly leading to an enriched deposition of GNPs in cancer cells compared to healthy tissue.

Recently we could show that localization microscopy [23] was a useful and precise tool to locate and quantify number and distribution of GNPs in cells [15]. Here we extend the field of application on SmartFlares [24, 25], RNA targeting nanogold probes that are aiming for the Her2 gene product in the endoplasmic reticulum (ER). From this product ErbB2 follows, one of the human epidermal growth factor receptors [26]. Cancer research has a focus on this special receptor that has shown to be overexpressed in 20–30% of breast cancers with poor prognosis [27] showing typical, nano-scaled spatial arrangements on the membrane and intra-cellular trafficking ([28]; Pilarczyk at al., manuscript submitted). As those SmartFlare probes were designed for RNA targeting while using the gold core just as a carrier for the dye, successful SmartFlare binding to the Her2 gene product in the endoplasmic reticulum would mean a GNP concentration in the ER around the cell nucleus. Thus, in tumor types where certain genes are considerably upregulated, the RNA of such genes could be targeted so that the GNPs accumulate in the tumor cells only, leading to an increased radio-sensitivity compared to non-tumor cells without extra-ordinary up-regulation.

In the following approach it will be shown for the example of Her2/neu related RNA that targeting of gene products enhanced in tumor cells might be a useful approach to obtain an increase in biological effectiveness of therapeutic irradiation. Using non-synchronized cells of a standardized cell line typically for breast cancer research [29], a model system was applied that may be close to a specimen of real tumor tissue.

## Material and methods

### SmartFlare setup

Her2/neu gene product targeting SmartFlare probes [24, 25] were bought from Merck Millipore as lyophilized powder. They consist of a 13 nm nanogold core conjugated to multiple copies of a double-stranded oligo-nucleotide, in which one strand (the “reporter strand”) bears a fluorophore that is quenched by its proximity to the gold core. When the nanoparticle comes into contact with its target RNA, the target RNA binds to its complementary “capture” strand and displaces the reporter strand. The reporter strand, whose fluorophore is now unquenched, fluoresces and can be detected [30]. SmartFlares were transfected into SkBr3 cell lines [29] according to manufacturer´s protocol. The capture strand is bound to the nanogold core with disulfide bonds and aims for Her2/neu gene products being in proximity to the ER. The SmartFlares accumulate close to the cell nucleus. The reporter strand labelled with a cyanine 5 (Cy5) can be observed separately and independently from SmartFlare and blank nanogold particles (detectable wavelength range from 488 nm—568 nm, while Cy5 fluoresces at 670 nm). The functionality of the SmartFlare protocol has been demonstrated in literature several times [15, 23, 31, 32]. This allows the conclusion that the functionally in the experiments here was successful, if the microscopic specimens showed Cy5 signals in the majority of the cells. Since the Cy5 labelled reporter strand is short, they diffused in the cytosol quickly so that the location of the RNA targeting could not be determined from the Cy5 dye distribution.

Unmodified GNPs with a diameter of 10 nm bought from Aurion Gold Sols (Aurion, Wageningen, The Netherlands) were transfected into the same cell lines as control probes (for details see [3]). After selecting the breast cancer cell lines (SkBr3 as cell line due to strongly overexpressing Her2/neu) the parameters of the experimental setup were optimized with respect to SmartFlare dilution, concentration and incubation time for different irradiation exposure doses.

### Cell culture and GNP incubation

SkBr3 cells were grown in McCoy’s 5A cell medium, containing 10% fetal bovine serum (FBS) and 1% penicillin/streptomycin. Cells were cultivated and maintained at 37°C in a humidified atmosphere at 95% air/ 5% CO_2_. After dissolving SmartFlares powder in 50 μl DNA free water, the solution was diluted with a ratio of 1:20 in 1xPBS. 8 μl of the diluted GNP solution was mixed with 1 ml cell medium and given to each cover slip (10^12^ particles per ml). Regarding unmodified GNP incubation, the same particle concentration was achieved by diluting 8 μl nanogold solution in 1 ml cell medium before adding to the cells. All chemicals used were of research grade. After 18 hours incubation time, the cell medium was changed and the probes were irradiated with doses of 0 Gy, 2 Gy and 4 Gy. Fixation was performed twice for 15 min each with a formaldehyde solution (4% in 1x PBS) 30 min after irradiation.

### Localization microscopy

Localization microscopy measurements were done for the optical investigation of the GNPs’ distribution as GNPs do not bleach under laser exposure but continue to blink even at maximum laser power [15, 33]. The frequency of incident photons is set in order to establish the resonance condition matching the natural frequency of the surface electrons (plasmon resonance [34]). Probes were measured with 491 nm and 561 nm two diode-pumped, solid-state lasers at 200 mW laser power. The instrument was equipped with an oil objective lens 63x/NA 0.7….1.4 (Leica, Wetzlar, Germany), an electron-multiplying charge-coupled device camera (1376 x 1040 pixels; Andor Technology, South Windsor, CT) and band-pass filters (525/50 nm for 491 nm excitation and 609/ 54 nm for 561 nm excitation).

The cells were selected visually. Stacks of 2,000 frames were acquired at an integration time of 50 ms each. To get comparable conditions, the cell nuclei were selected in such a way that the image section was taken at the largest diameter. For quantitative image evaluation, in-house programs were applied (for details see Grüll et al. [35], Kaufmann et al. [36], Müller et al. [37], Stuhlmüller et al. [38]).

### Spinning disk microscopy

Three dimensional z-stacks with a z-resolution of 150 nm were taken at Nikon Imaging Center at Bioquant Heidelberg, using a Perkin Palmer ERS6 spinning disk microscope. All stacks were measured with a Nikon Plan Apo λ 100x/NA 1.45 oil immersion objective and a Hamamatsu C9100-02 EMCCD camera (1000 x 1000 pixel, 8 μm pixel size). Lasers of 405 nm, 488 nm, 561 nm and 640 nm excitation wavelength were used for fluorochrome activation such as DAPI DNA counterstain and Alexa stained antibodies. For green (527/55), blue/red (455/60 and 615/70) and far red (485/60 and 705/90) dual pass filters were available. The number of pictures taken per stack was picked manually in order to measure individually over the whole cell nucleus. After microscopy image stacks were de-convolved with Huygens Remote Manager software, to improve picture quality.

### γH2AX foci analysis

Radiation-induced lesions are mainly represented by DNA double-strand breaks (DSBs) [39]. To present day DSB repair provides the closest correlation available with radio-sensitivity. H2AX, a variant form of the histone H2A, undergoes extensive phosphorylation at the DSB, creating γH2AX foci that can be visualized by immunofluorescence. As there exists a close correlation between γH2AX foci and the number of DSBs and between the rate of foci loss and DSB repair, foci analysis has shown to be a sensitive assay for quantifying a dose deposition on the cell [40, 41].

To show a GNP induced effect on the dose deposition and therefore ruling out the possibility of a simple “raining down” effect of the GNPs onto the cells surface and therefore proof GNP uptake by the cells, foci counts, locations and distributions were analyzed. Therefore, three dimensional z-stacks have been imaged with a Perkin Palmer ERS6 spinning disk microscope provided by Nikon-Imaging-Center, Bioquant Heidelberg. For SkBr3 cells the dose enhancement compared to control probes and unmodified GNP treated cells was quantified via foci analysis while for each analysis a minimum of 50 cells was measured and quantified. Therefore formaldehyde (prepared freshly from paraformaldehyde) fixed cells on cover slips were treated with 0.2% Triton X in 1xPBS+Mg/Ca and BSA blocking solution. Cells were stored overnight with γH2AX Anti-Phospho-Histone (Sigma Aldrich; 1:250 in BSA) at 4°C. Secondary antibody solution (anti mouse) with the same concentration was incubated over the second night at 4°C before DAPI stain (1:20,000) followed by fresh formaldehyde fixation and sealing the cover slips with enamel. For comparison of foci counts in different experimental runs, a t-test (Welch test) [42] was applied.

### Contextual interactive light and electron microscopy (CILEM)

In order to perform transmission electron microscopy (TEM) from the same cell samples as examined by light microscopy, a novel technological approach called contextual interactive light (and) electron microscopy (CILEM) was developed and applied. Details of CILEM and its potential for application in microscopic investigations will be published elsewhere (Pilarczyk et al., manuscript submitted). Briefly: After light microscopy data acquisition the specimens on glass slides were unmounted by swelling the ProlongGold embedding media in PBS at 40°C for 48 hrs. The lift off process removing the glass slides with the adhering cells from the object carrier was done in the swelling medium. This environment totally excludes the presence of liquid to air phase borders which are the main reason for micro-displacements and mechanical specimen loss. Further, the release by gravitation forces over a long period minimizes local liquid convections. Also, specimen deformation as usual during mechanical unleashing processes does not appear because the gravitation force is homogeneous over the entire specimen. The released cover slides were fixed in 7% glutaraldehyde in PBS at 4°C for 18 hours and washed 8 times for 4 min each in PBS. For contrast generation 1% osmium tetroxide in ultrapure water was applied for 1 h. The specimens were desiccated by an ethanol series (10 minutes each for once 25%, 50%, 75%, 90% and three times 100%) and immersed in Epon (2 ml each) at 40°C for 12 hrs. After changing the Epon resin three times for one hour, the specimens were mounted on top of gelatin capsules filled with resin. After polymerization at 60°C for 16 hrs the specimen were blasted from the glass carrier by plunging the total specimen mount of gelatin capsules sticking to cover slides with cell monolayer locating at the glass to resin interface in liquid nitrogen. Rubbing the splattered glass manually from the ultra-cold resin leaves the cell monolayer at the resin surface. After post curing the resin pillars at 60°C for 24 hours and mounting in an ultracut holder (Leitz, Wetzlar, Germany) ultra-thin slices of 60 nm were cut with a diamond knife in an Leica UltraCut apparatus (Leitz, Wetzlar, Germany) and put on Formvar folia coated carrier grids (Plano, Wetzlar, Germany). The slices were positive-stained for 10 minutes with uranium acetate (Serva, Heidelberg, Germany) 1% (m/v) in ultrapure water. TEM imaging was performed on a Zeiss 10A transmission electron microscope (Zeiss, Oberkochen, Germany) with a varying magnification of up to 125,000 fold.

### Magneto-experiments

The influence of a magnetic field between 0.2 T and 3.0 T on the dose enhancement has come into research focus as hybrid MRI/irradiation setups for clinical use have been introduced. It has been shown that magnetic field effects are most prominent in the field range of 0.75 to 1.5 T [43]. In order to investigate the influence of a magnetic field on the GNP distribution and the achieved dose enhancement, irradiation experiments were conducted with and without a simultaneously applied magnetic field. Therefore, a petri dish containing SkBr3 cells was positioned between the pole shoes of an electromagnet EM1 (Magnet-Messtechnik Jürgen Ballanyi, Germany). SkBr3 cells were cultivated following the protocol as described above. Irradiation experiments were done using a 6 MV photon beam of a VersaHD linear accelerator (Elekta AB, Stockholm, Sweden).

The magnetic field strength was about 980 mT which well correlates to the best effectiveness reported in [43]. It was adjusted with a Hall probe. Reference cells were not irradiated but were only exposed to the magnetic field. SkBr3 cells were irradiated with doses of 0.5 Gy, 2 Gy, and 4 Gy respectively with a dose rate of 400 MU/min. Formaldehyde fixation (as described above) took place 30 min after irradiation.

## Results

**Fig 1:**
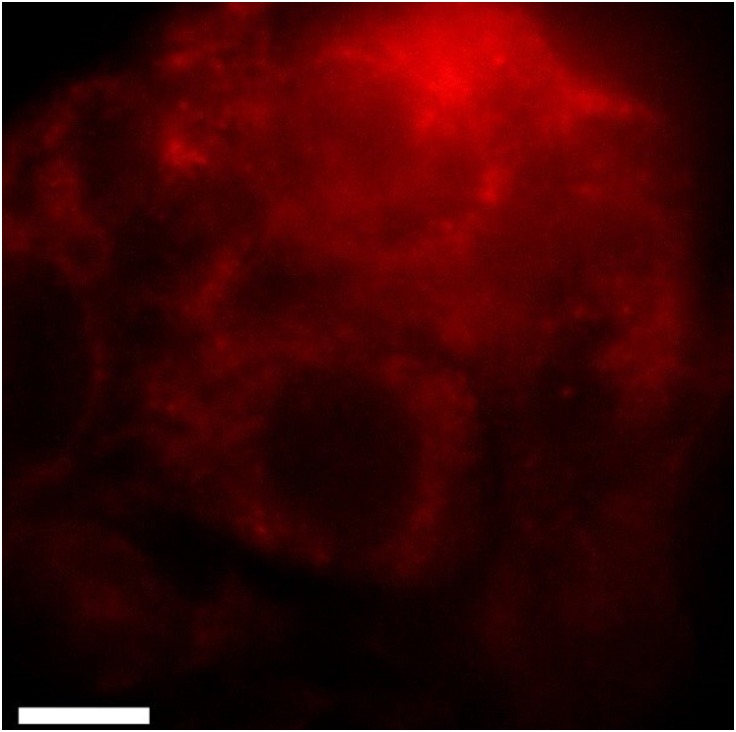
Epifluorescence image of SkBr3 cells transfected with SmartFlares illuminated with a 671 nm laser. While Cy5 signals can be clearly seen in the cytoplasm of the cells, cells treated with no or unmodified GNP are almost not visible due to the very low auto-fluorescence at 671 nm (not shown). The scale bar equals 20 μm.

Before investigations on the GNP distribution inside the cell and a quantification of the dose enhancement were made, the SmartFlare quenching concept was examined in order to verify a successful quenching of the release strand. Cy5 detection via epifluorescence microscopy (100x/ NA 1.44 objective, pixel size 64.5 nm) shows the release of the reporter strand, stating a successful SmartFlare binding around the cell nucleus. Neither for untreated probes nor for cells treated with unmodified GNP of different sizes any signal but noise could be detected at 671 nm, in contrary to the released Cy5 reporter strands as can be seen in Fig 1.

Investigations on the GNP distribution itself were done via localization microscopy. After illumination with a 491 nm and a 561 nm excitation laser wavelength the signal count for probes transfected with unmodified GNPs, SmartFlare probes and untreated control probes was obtained in order to quantify and compare mentioned probe setups. For each setup a minimum of 50 cells were measured.

A peak of the signal count dependent on the incubation time was found at about 18 h for both wavelengths and for all three probe setups. Incubation times of 6 h, 12 h and 18 h showed an almost linear dependence of the measured signal count. For longer incubation times the signal count starts to drop again leading to the assumption that the GNPs tend to be transported out of the cells again subsequently to transfection. These findings were in good agreement to our recent results [3, 15] in which the time dependent uptake and loss of unmodified GNPs of 13 nm and 25 nm was evaluated by absolute counting of GNP point signals [15].

Regarding the dependence on the applied amount of GNP solution no peak could be found. For 2 μl, 4 μl, and 8 μl the signal count increased with the applied GNP concentration. However, SmartFlare amounts higher than 8 μl did only lead to a slight increase of the detected signal count. For higher amounts signal counts of localization microscopy measurements suffered from a higher standard deviation.

Subsequent bleaching experiments with localization microscopy at maximum laser power of 200 mW show that the GNP blinking signal remains stable even for exposure times longer than 40 min. Comparing image stacks taken at the beginning of the bleaching measurements and at the end lead to very similar GNP signal counts detected via localization microscopy while background signals on the other hand are bleached out after a short moment due to the high laser power.

**Fig 2:**
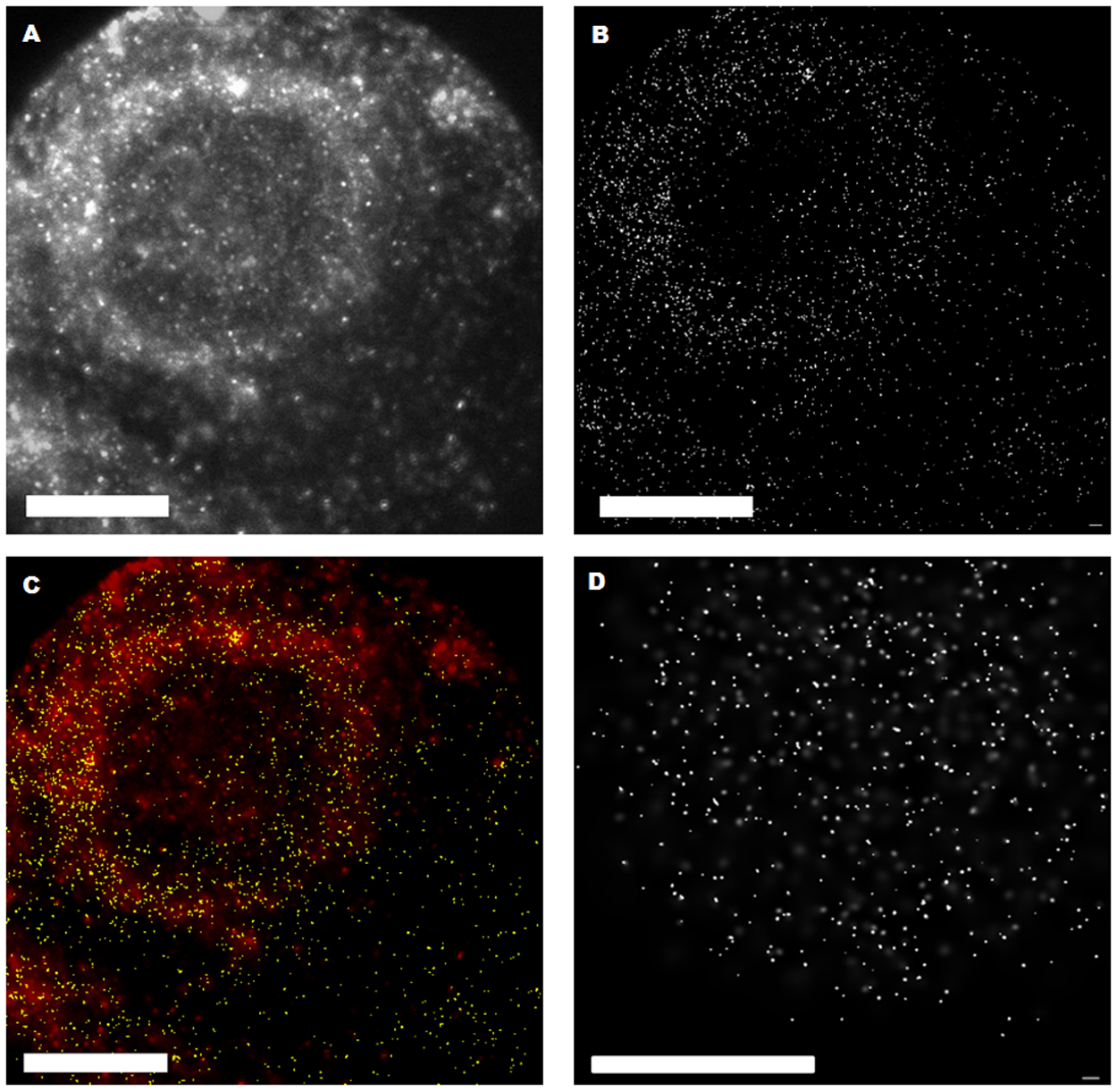
Localization microscopy images of a SkBr3 cells. Illumination at 561 nm transfected with 8 μl SmartFlare solution after 18 h incubation time with the characteristic ring like shape (A-C). Wide-field overview (A) and localization microscopy image (B) with points representing the loci of blinking events. Merged image (C) of wide-field overview (red) and detected GNP signals (yellow) of the pointillist localization microscopy image. Localization microscopy image of a cell treated by unmodified GNP for comparison (D). The scale bars equal 20 μm each.

While SPDM images of the control probes and unmodified GNP show no characteristic conglomeration (e.g. unmodified GNP signals spread over the whole cytoplasm), up to 10% of the SmartFlare targeted cells end up accumulating the GNP cores in a characteristic ring-like shape around the cell nucleus (see Fig 2). For those cells, about 80% of the signals detected with localization microscopy are found within the ring area, while for other cells there could only be detected a statistical GNP distribution over the whole cytoplasm. If a measured cell shows this characteristic ring like shape at one wavelength it was also observed for the second wavelength.

**Fig 3:**
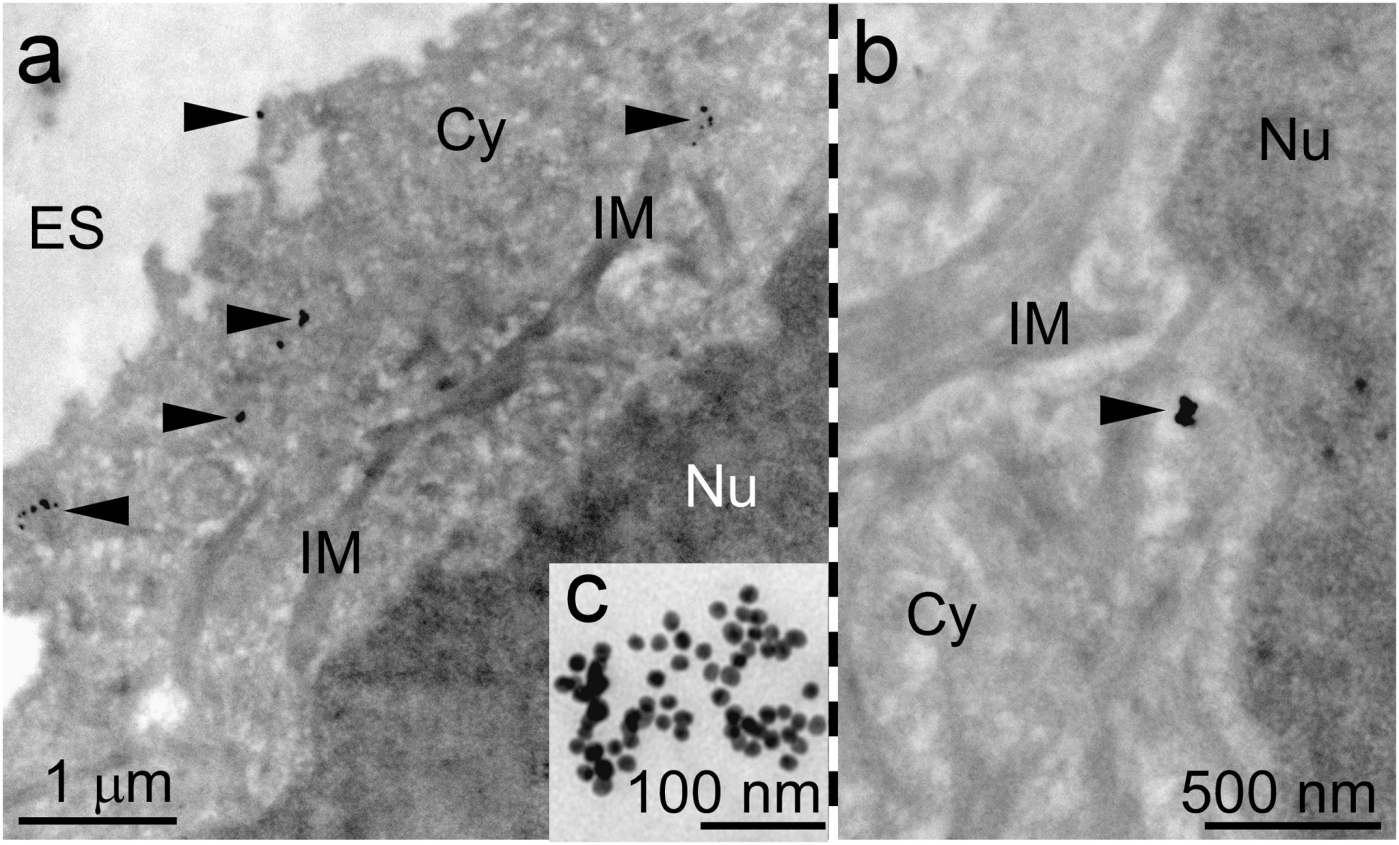
Transmission electron microscopy images show GNPs being included into the cytosol but excluded from the lumina of intracellular vesicles, microbodies, the golgi apparatus and the endoplasmic reticulum. Further GNPs are preferentially accumulated in groups of varying extend. A. Overview of a group of GNPs (black arrow) inside the cytosol (Cy). The extracellular space (ES) and the nucleus (Nu) do not carry any GNP accumulations. B. Locality in the cytosol with a local GNP accumulation in the vicinity of components of the intracellular membrane apparatus (IM). C. Insert showing the homogeneous size of SmartFlares in a cluster.

Table 1 enlists the average signal count for unmodified GNP and SmartFlare transfection for both 491 nm and 568 nm. These two wavelengths were chosen in order to eliminate that cells show autofluorescence signals similar to GNPs (for details of such autofluorescence see [44]). One can see that on the one hand there are slightly more signals to be found at 491 nm than at 561 nm. A reason for this might be found when considering absorption spectra of GNPs. The maxima of the fluorescence peaks for GNPs of about 10 nm diameter (at about 518 nm) are slightly closer to 491nm than to 561nm [14]. Although the size distribution is rather homogenous for SmartFlares (see Fig 3), it cannot be excluded that size variations contribute to this difference. As the peak is approximately symmetric a larger distance in wavelength goes hand in hand with lower absorption and therefore less blinking signals. On the other hand the standard deviation (SD) is bigger for 491 nm, compared to 561 nm laser exposure which may also be due to size variations and detection sensitivity differences. Nevertheless, comparing the mean values of counts for both wavelengths within the standard deviations for both unmodified GNPs and SmartFlares, they very well coincide so that autofluorescence signals can be neglected.

**Table 1:** Localization microscopy signal counts for unmodified GNP, SmartFlares. For each wavelength of 491 nm and 561 nm standard deviation and the error of the mean are given.

	491 nm	SD	Error of the mean	561 nm	SD	Error of the mean
Unmodified GNP	9,348	2,708	1,104	8,497	1,990	871
SmartFlares	16,912	4,843	1,218	13,820	3,723	941

The ratio of signals in proximity of the cell nucleus to the total signal count is about the same for both wavelengths. For those cells which show the characteristic ring structure around the cell nucleus, the signal count within the ring area is up to 80% of the total signal counts detected. Averaged over ring showing cells, the signal ratio is about (68% ± 12%) for 491 nm and (73% ± 8%) for 561 nm which also coincides within the error ranges.

In order to study the localization of SmartFlare GNPs after activation in the cell, transmission electron microscopy (TEM) was performed with the same specimens as being used for microscopy. Applying CILEM allows the analysis of the same cell ensemble by light microscopy on glass slides and TEM on grid carrier (Pilarczyk et al., manuscript submitted). The TEM data indicated preferred arrangements of SmartFlare GNPs between membranous structures near the cell nucleus which could be assigned to the endoplasmatic reticulum. In Fig 3 a typical result of a TEM image is presented. The overview image indicates that the morphology of the cells is well maintained in CILEM. The enlarged image section shows the typical arrangements of GNPs in vicinity of components of the intracellular membrane apparatus while the insert highlights the homogenous size distribution of GNPs within a clustered distribution.

After evaluating the GNP distribution inside the cell and quantifying the influence of modification on GNPs on accumulation in target areas of the cell, the influence of GNP distribution on the dose enhancement was investigated via γH2AX Foci-Analysis.

Irradiation experiments were executed for SkBr3 cell line at doses of 0 Gy, 2 Gy and 4 Gy. For the detection of γH2AX Foci, the cells were measured with a Spinning Disk Microscope giving 2D projection images of the Foci (see Fig 3).

**Fig 4:**
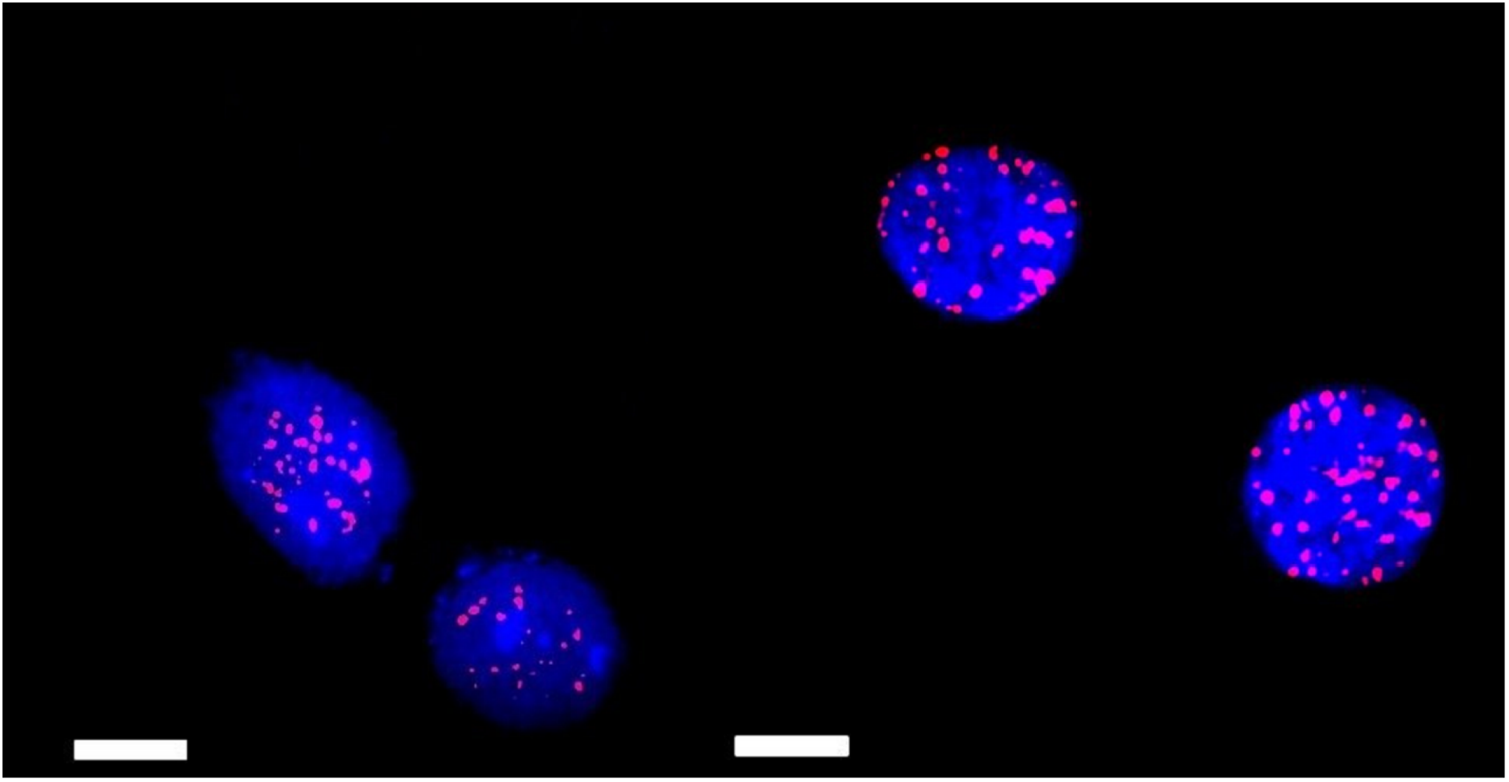
Analysis of γH2AX foci (red) for different SmartFlare concentrations incubated for 18 h on SkBr3 cells (cell nuclei are stained in blue by DAPI) after irradiation of 4 Gy: control probe (left), and 8 μl SmartFlare transfected (right). The scale bars equal 20 μm.

**Fig 5:**
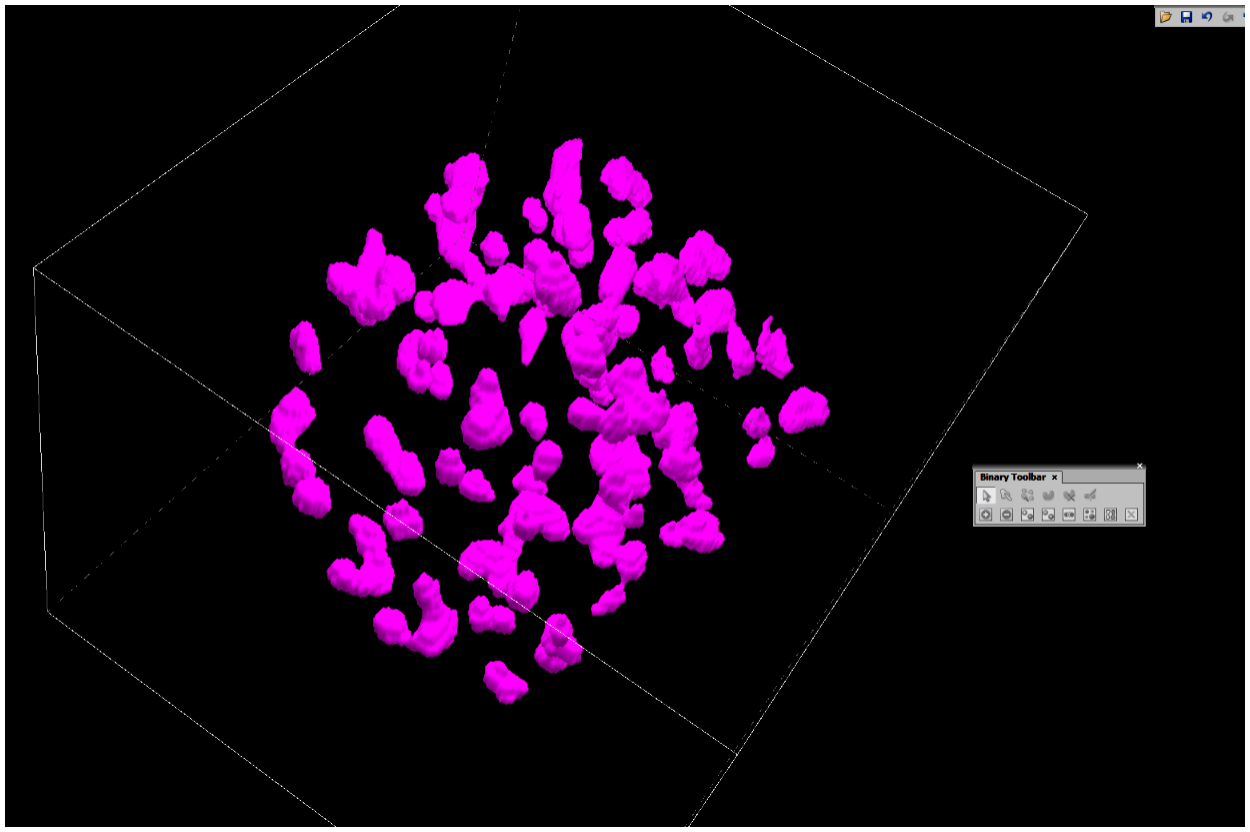
3D image of fluorescently labelled γH2AX foci (pink) in a SkBr3 cell nucleus after irradiation of 4 Gy and incubation with 8 μl SmartFlare solution. The foci are represented with NIS-Elements. The cube’s edge length equals 20 μm. No special foci arrangement or distribution could be detected, independently from dose or GNP concentration.

As can be seen in Fig 4, the amount of foci is that high at doses of 4 Gy, the signals might overlap in 2D view. For this reason three dimensional z-stacks were measured in order to count them over the whole cell and also to measure the location of the foci inside the cell nucleus. The z stacks were analyzed with the program “NIS Elements” at Nikon-Imaging-Center, leading to the result that the Foci seem not to have a characteristic, but a statistical distribution over the whole cell nucleus (see Fig 5).

**Fig 6:**
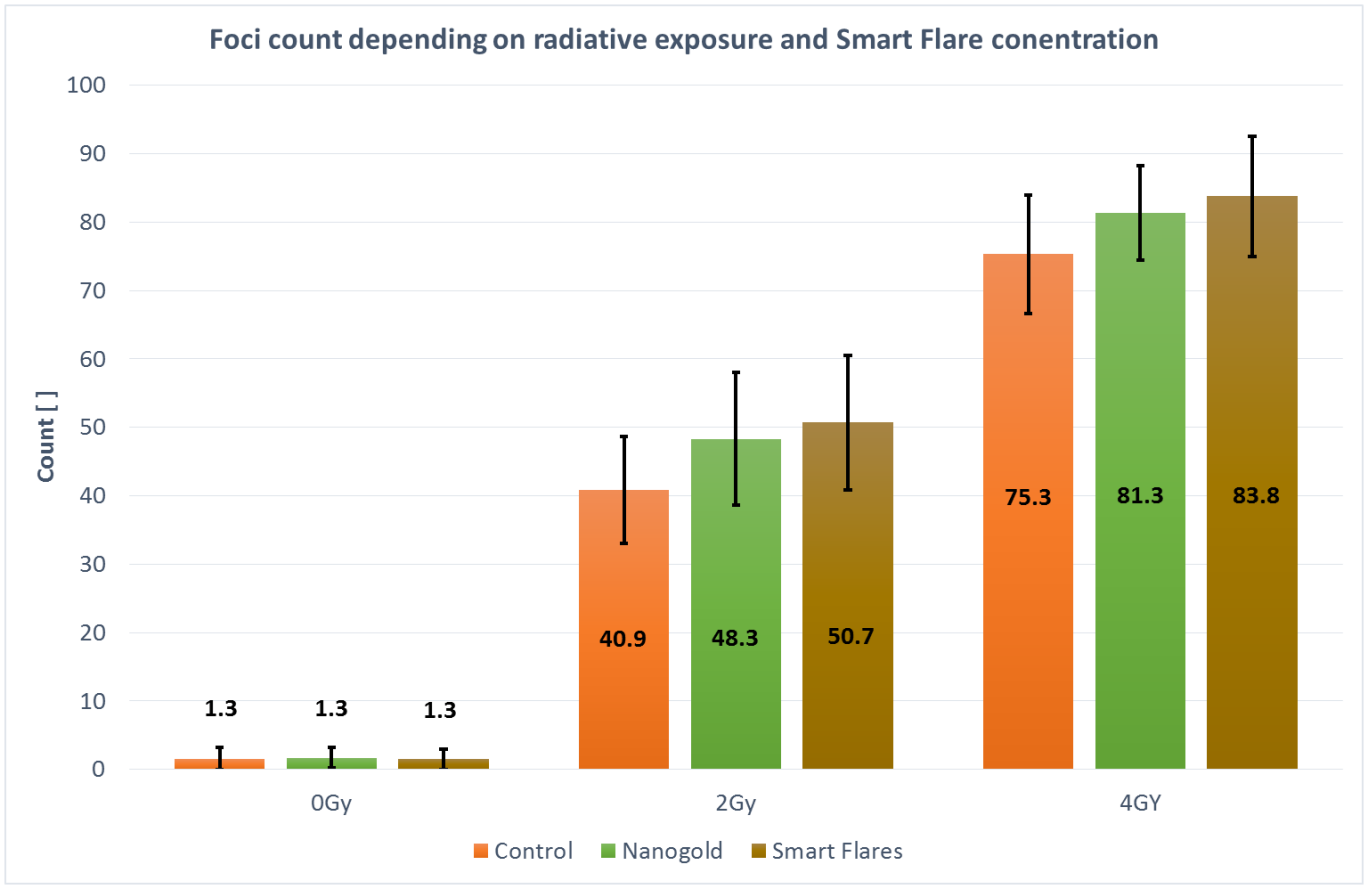
Comparison of the averaged detected foci count for control (orange), unmodified GNP (green) and SmartFlare probes (brown) at 0 Gy, 2 Gy and 4 Gy. The standard deviation is highlighted as black arrow line. A significance level of 0.05 was used for all statistical tests. In Table 2 (below) the significance levels are presented which would be required that the significance disappears. These values have to be seen in relation to 0.05 if one compares the histograms in this figure.

The number of foci per SkBr3 cell was determined with NIS elements in order to compare the dealt dose for a given irradiation exposure and setting. Fig 6 shows the different foci counts comparing the impact of 0 Gy, 2 Gy and 4 Gy on control probes, unmodified GNP and SmartFlare treated cells. While at 0 Gy no difference is perceivable, unmodified GNP and SmartFlare foci counts are significantly higher for both 2 Gy and 4 Gy doses. For the mentioned settings the foci analysis has been repeated four times, all resulting in achieving maximum foci counts for SmartFlare treated cells. Furthermore—in order to double check the validity of the software—the foci were counted by hand yielding to the same results. Since the program applied a constant threshold onto the foci of all doses, the absolute foci counts turned out to be slightly higher when counted by hand. The relative values between control, unmodified GNP and SmartFlare treated probes were almost exactly the same though, implying a dose enhancement despite of a relative high standard deviation of the single measurements.

Comparing SmartFlare and unmodified GNP treatment on the one hand, it could be shown that SmartFlare foci mean counts were all higher for every single of the four independently executed foci analysis as can be seen in Fig 6.

P-values obtained by the results of the foci analysis are listed in Table 2 for different doses. Statistical evaluation with the Welch’s test yield p<0.01 for comparing the foci counts of both SmartFlares and unmodified GNPs to the control probes thus stating a high significance of both kinds of GNP treated cells compared to the control probes. The SmartFlare treated measurement line shows a more than 10 times smaller p-value than unmodified GNP treated cells for both 2 Gy and 4 Gy. However, comparing SmartFlares with unmodified GNP counts leads to a p-value higher than 0.05.

**Table 2:** Comparison of the p-values according to Welch’s test. While non irradiated probes show p-values much higher than 0.05, unmodified GNP and SmartFlare treated probes differ highly significantly (p<0.01) from corresponding control probes. P-values for measurement results comparing SmartFlares and unmodified GNPs treated probes are higher than 0.05. This table has to be seen in correlation to Fig 6.

	Unmodified GNP to control	SmartFlares to control	SmartFlare to Unmodified GNP
0 Gy	0.432	0.500	0.428
2 Gy	0.000281	0.00000537	0.165
4 Gy	0.000824	0.000159	0.114

Further investigations were made on the foci volume, the nucleus membrane distance of the foci and their ratio to the total nucleus volume (see Table 3). It turned out that both average foci volume, as well as membrane distance remain constant and are therefore independent of irradiation and GNP/SmartFlare concentration and treatment. This is in good correlation to recently obtained localization microscopy measurements of γH2AX foci formation and dissolving during DNA repair (Hausmann, Scherthan et al., manuscript submitted). As the number of the foci increases with dose and GNP treatment while the individual foci volume stays constant, the ratio of the volume of all foci to the total volume of the cell raises proportional to the count.

**Table 3:** Analysis of foci volume and arrangement within the cell nucleus from top line to bottom line: Foci count of the fourth experiment analyzed with NIS elements; average membrane distance of the foci in [%] compared to the maximum possible radius within the nucleus; average foci volume in arbitrary units; volume ratio of all combined foci to the nucleus volume; note that the two values in brackets faced a very high standard deviation and are—Due to the low foci count—Not used for any conclusion even though fitting well to the other values.

	0 Gy	0 Gy	2 Gy	2 Gy	2 Gy	4 Gy	4 Gy	4 Gy
	Control	Smart Flares	Control	Unmodif. GNP	Smart Flares	Control	Unmodif. GNP	Smart Flares
Foci Count (NIS-Elements)	1.9	1.7	46.1	53.0	56.2	68.4	68.7	74.2
Mean membrane distance [% nuclear radius]	(31)	(28)	30	30	42	36	40	31
Volume [a.u.]	63	64	55	63	72	65	61	70
Volume ratio [%]	~0	~0	4.4	5.2	5.4	6.5	6.7	6.9

### Investigations on the influence of a simultaneously applied magnetic field

Specific radiation techniques might be combinable with a simultaneously applied magnetic field. It has been shown that special GNP setups can have large magnetic moments originating from the particle’s Au 5d band, containing a high number of holes. Bartolome et al. could show that GNPs can be strongly paramagnetic when grown on S-layers via reduction with dimethylaminoboran hydride [45]. Even for bare GNPs doped with Gd high magnetic moments could be measured [46]. Although these experiments have been done with GNPs with a diameter of just 2nm, it still raises the question, whether an applied B-Field during irradiation has an influence on the dose enhancement and the GNP distribution within the cells. This question might also be relevant for recently introduced hybrids of MRI and radiation therapy [47]. The results for B = 980 mT are given in Table 4. Neither an influence on the number of foci, nor on the foci volume, the foci distribution or the nuclear membrane distance were measured (results of Welch’s test not shown).

**Table 4:** Results of magnetic field probes after conducted γH2AX foci analysis: No significant impact of the magnetic field could be detected (standard deviations in brackets).

unmodified	B = 0 T	B = 980 mT	SmartFlares	B = 0 T	B = 980 mT
0 Gy	-	3.9 (1.2)	0 Gy	-	2.1 (1.4)
0.5 Gy	7.9 (2.1)	7.6 (2.0)	0.5 Gy	7.7 (1.4)	8.0 (2.7)
2 Gy	52.6 (17.2)	56.4 (14.9)	2 Gy	57.7 (19.0)	58.2 (16.2)
4 Gy	66.1 (23.8)	65.2 (21.2)	4 Gy	73.7 (20.0)	75.2 (21.9)

## Discussion

Here we report as a proof of principle and with the help of localization and Spinning Disk microscopy as well as transmission electron microscopy that GNP modification can lead to specific GNP distributions and accumulations at target regions in vicinity of the endoplasmatic reticulum of SkBr3 cells. About 10% of the cells evaluated by localization microscopy showed a characteristic SmartFlare accumulation around the cell nucleus containing roughly 80% of all the detected localization microscopy signals. Although the damaging effects measured by γH2AX foci counting was not significantly different for both types of GNPs applied, the accumulation of SmartFlares around the cell nucleus and in vicinity of the endoplasmatic reticulum may reason that a tendency of increased damaging was found for SmartFlares. Transmission electron microscopy (TEM) is capable to spatially resolve the GNP intracellular distribution and local accumulation. In difference to the spontaneous sphere accumulation under extended storage times in vitro, the local accumulation in vivo must be considered as a result of the cytosolic organelle tracking activity. TEM could not resolve any membrane coating on GNP accumulations in the cytosol. This might be due to the staining procedure or be a result of the preceding mounting for light microscopy. In any case the finding of GNP accumulations as a result of an active cell tracking process indicates the distribution of GNPs being non-random but under the control of the GNP housing cell. It could be shown that modified GNP accumulation leads to a γH2AX foci count that is highly significantly increased by a factor of up to 1.2 compared to untreated cells. This increase is not generally the case for other nanoparticles like Gadolinium as investigated by Porcel et al. or Stefancikova et al.. Despite observing a decrease of the clonogenic survival fraction they did not detect increased foci numbers as compared to irradiation without nanoparticle incorporation [48–50]. The drop in the survival curves due to nanoparticle application is in accordance with our results previously published in this journal [3]. As Porcel et al. and Stefancikova et al. used Gd particles with a size smaller by a factor of up to 3, there is further research to be done on the question of how these nanoparticles interact with the different functional compartments of a cell beyond direct DNA damaging. The factor of 1.2 is in accordance with other publications on GNP caused dose enhancement [1, 20–22]. This is once confirmed during a second subsequent experimental run during investigations on the influence of the magnetic field on GNP distribution and dose enhancement. Although prior publications state that small GNPs of 2 nm diameter may take up a large magnetic moment [45, 46], no B-Field influence could be found for GNPs with a diameter of 13 nm.

Due to the three dimensional bulb shape of the epithelial cell nucleus it might be possible that GNPs slide down along the nucleus bulb ending up at the ground level edge of the nucleus and the cytoplasm. Those additional GNPs might be detected by localization microscopy without being part of the cellular uptake mechanism (for comparison see [15]). Such a simple GNP “rain down” effect onto the cell’s surface had to be considered and was ruled out by TEM imaging where the mechanically cut slices showed a clear localization within the ER membrane structures. Furthermore all localization microscopy images showed evenly distributed unmodified GNPs over the cytoplasm, stating that the rain down effect could not be detected for unmodified GNPs. Therefore it is not feasible to consider a significant part of the detected SmartFlare ring signals to originate from above the cell’s surface. Epifluorescence microscopy images of the Cy5 signal also show the successful SmartFlare binding [25] supporting the assumption that the aspect of a simple rain down effect making up a significant part of the ring signals can be neglected.

Although only 10% of the SmartFlare transfected cells showed a different distribution from unmodified GNPs, every set of foci analysis showed higher values for the absolute foci count of SmartFlare transfection compared to unmodified GNPs.

The low ratio of detected rings combined with the higher foci count might be a hint for the potential that could be used for a controlled accumulation in proximity to the cell nucleus (or inside). The main part of the SmartFlares, if not binding to the RNA targets, might act the same way as unmodified GNPs spread all over the cytoplasm. This might explain that the foci analysis was not significantly different between SmartFlares and unmodified GNP but showed a pronounced tendency to increased values.

The effect that the detected damage was not significantly different between SmartFlare and GNP application raises the question whether SmartFlares have really targeted the corresponding RNA. The Cy5 results support the activation of SmartFlares. However, the specific targeting could additionally verified by the application of SmartFlares with scrambled sequences as controls. So far such SmartFlares are not available and for their design a complex bioinformatics approach is required. This will be subject of future designs and syntheses.

A low energy electrons’ mean free path though might not be large enough to reach the cell nucleus from most parts of the cytoplasm [9, 10]. If GNPs are evenly distributed in the cytoplasm, most of the measured effect may then not be by damage on DNA in the nucleus. If the Auger electrons directly hitting DNA strands in the cell nucleus were the main source of the dose enhancement, investigations on the three dimensional foci distribution would probably not have led to an even foci distribution over the whole cell nucleus. Possibly the foci density would decrease towards the nucleus center due to limited Auger emitter range. Thus the question arises whether there are other kinds of interactions between GNPs and the cell on a rather indirect functional chain resulting in a higher foci count and further investigations have to be done on that matter.

An important point is the fact that localization microscopy could only detect two kinds of cells treated with SmartFlares: those cells with the majority of the signals around the nucleus and those with a completely even distribution over the cytoplasm. There were no cells found with a slight or notable increase of signals around the nucleus. It rather seems that the successful SmartFlare binding depends on the cell itself, even when being on the same cover slide and being treated with the very identical SmartFlare solution. This might lead to the question whether all cells show the same over-expression of Her2/neu or whether the cell cycle may play a role for GNP absorption and efficacy. For instance, it could be shown that GNPs bound to glucose molecules treating prostate cancer cell line DU-145 had a crucial effect on the individual cell’s cell cycle by significantly decreasing tumor protein p53 and arresting cells in radio sensitive G2 phase [51].

Regarding our experiments, SkBr3 cells were not synchronized prior to GNP transfection in order to gain a realistically quantification of GNP distribution and GNP caused dose enhancement in correlation to application in radiotherapy. Nevertheless it might be mentioned that synchronized cultures may be helpful for investigations of the mechanisms of GNP uptake and localization. This was not the scope of this article and will be addressed in future projects. Further investigations have therefore on the one hand to be made on the question whether cell synchronization would lead to an increase of the cell ratio accumulating GNPs around the nucleus and/or the γH2AX foci count. On the other hand further investigations will be necessary concerning the matter, in what way GNPs ending up in the cytoplasm might influence other crucial parts of the cell than DNA, such as mitochondria, lysosomes or the functional chain of protein dynamics with respect to repair processes.

The internalisation into the cell was not studied in detail here. Since the SmartFlares were available for several RNAs, endocytosis should not be a limitation. In the TEM images we found individualized particles and linear arrangements of particles that do not speak for clustering in vesicles. Nevertheless control of particle uptake and active membrane transfer for instance via appropriate peptides will be subject of future projects.

In conclusion, once more localization microscopy is confirmed to be a reliable and crucial tool for high resolution quantitative investigations on the distribution and location of GNPs inside cells (see also [15]). The novel approach of CILEM allows the application of TEM to the same samples prepared for localization microscopy so that addition super-resolution image information can be obtained. Here we make a first attempt to get a full quantitative analysis of testing modified GNPs by combining localization microscopy and TEM for GNP localization and quantification and by three dimensional γH2AX foci analysis for quantifying the corresponding dose enhancement. As it could be shown in principle, that GNPs targeting special regions of a cell, can be precisely imaged at any given time during incubation, GNP constructs that have yet just be simulated or theoretically investigated, are now to be tested under lab conditions. This offers new perspectives in analyzing and thus investigating interactions of targeting and non-targeting GNPs in cells in order to better understand mechanisms behind increasing biological effectiveness for therapy purposes. Although the specific incorporation in tumor cells is a matter of a long debate, our approach of specific targeting of gene products considerably up-regulated in tumor cells only may be a novel approach toward solving this still open methodological challenge.
